# Irinotecan induces intestinal atrophy and compromises duodenal motility and mucosal permeability: in vivo and in vitro evaluations

**DOI:** 10.1007/s00280-026-04965-y

**Published:** 2026-10-26

**Authors:** Nathalie Arendt, Tania Payo-Serafín, Hans Lennernäs, Femke Heindryckx, Markus Sjöblom

**Affiliations:** 1 Department of Medical Cell Biology, Uppsala University, BMC, PO Box 571, Uppsala, SE - 751 23 Sweden; 2 Institute of Biomedicine (IBIOMED), Universidad de León, León, Spain; 3 Department of Pharmaceutical Biosciences, Uppsala University, Uppsala, Sweden

**Keywords:** Intestinal barrier function, Mucosal permeability, Motility, Chemotherapy, Cancer, Viability, Mucositis, Irinotecan, CPT11, SN-38

## Abstract

**Purpose:**

Intestinal toxicity is a major adverse effect of irinotecan (IRT) chemotherapy, and effective preventive treatments are lacking. This study evaluated the impact of IRT on (i) duodenal motility and mucosal barrier function, (ii) histopathological changes along the small intestine and colon, and (iii) faecal water content, to improve understanding of IRT-induced intestinal injury and support development of future therapeutic strategies.

**Methods:**

Anaesthetized Sprague–Dawley rats underwent in situ duodenal perfusion to quantify the effects of intravenous (i.v.) IRT on duodenal motility and blood-to-lumen clearance of ³H-mannitol (paracellular route). In separate experiments, intestinal toxicity was induced by intraperitoneal (i.p.) IRT. After 72 h, rats were sacrificed and the small intestine and colon collected for histopathological evaluation. Body weight and faecal water content were assessed. Additionally, the effects of IRT and its active metabolite SN-38 on cell viability were examined in HCT116 cells.

**Results:**

IRT (60 mg/kg, i.v.) markedly increased duodenal motility (+ 277%) and mucosal paracellular permeability (+ 85%) immediately after administration. At 72 h after administration, IRT (180–200 mg/kg, i.p.) reduced villus length in the duodenum (− 52%, − 45%) and jejunum (− 37%, − 33%), with no significant effect in the ileum, and increased jejunal crypt depth (+ 48%, + 41%). In addition, IRT (i.p.) elevated faecal water content (+ 19%, + 16%) and reduced body weight (− 10%, − 13%). In vitro, IRT and SN-38 decreased HCT116 viability in a concentration-dependent manner, with IC₅₀ values of 19.4 µM and 12.8 nM.

**Conclusion:**

IRT increases duodenal motility, disrupts mucosal barrier integrity, induces diarrhoea, and causes small-intestinal injury. SN-38 shows substantially higher cytotoxic potency. These findings provide mechanistic insight into IRT-induced intestinal toxicity and may inform development of strategies to mitigate gastrointestinal side effects in cancer patients.

## Introduction

Cancer is one of the leading causes of death worldwide and resulted in almost 10 million cancer-related deaths in 2020, a number estimated to increase to over 16 million by 2040 [1]. Chemotherapy is a cornerstone of cancer treatment, serving as the first-line therapy for many malignancies [2]. However, while these cytotoxic agents effectively target rapidly dividing tumour cells, they also affect proliferating non-tumour cells, particularly epithelial cells in the gastrointestinal (GI) tract and hematopoietic cells, leading to significant side effects, such as diarrhoea, vomiting, bone marrow suppression, hair loss, shortness of breath, and fever, which reduces overall quality-of-life [3, 4]. It is believed that the incidence of GI adverse events strongly contribute to treatment discontinuation and suboptimal adherence for anticancer therapy.

Irinotecan (IRT, CPT-11), a semi-synthetic derivative of camptothecin, is a key chemotherapeutic agent used to treat various neoplasms, including colorectal, gastric, pancreatic, and ovarian cancers [5]. As a broad-spectrum cytotoxic drug, IRT exerts its antitumor activity primarily by its active metabolite SN-38 (~ 95–99%), not the parent compound, inhibiting topoisomerase I (Topo1), an enzyme involved in DNA replication and transcription [6–8]. By stabilizing the Topo1-DNA cleavage complex, IRT, or mainly SN-38, induces DNA strand breaks, ultimately triggering apoptosis in dividing cells. While Topo1 inhibition is well established as IRT treatment’s primary mechanism, emerging evidence suggests that IRT and mainly SN-38 may also exert anti-proliferative effects through Topo1-independent pathways [5].

Despite its efficacy, IRT treatment is frequently limited by dose-dependent toxicities, particularly GI complications and neutropenia. Among these, diarrhea is a major dose-limiting side effect, occurring in between 40 and 100% of patients [9]. Chronic or severe diarrhea may not only necessitate dose reductions, treatment discontinuation and significantly decrease the patient´s quality of life, it can also pose a life-threatening risk [9]. Although IRT remains a cornerstone of cancer therapy, further research into its toxicity mechanisms is needed to optimize its clinical utility while minimizing adverse effects.

The prodrug IRT is hydrolyzed into SN-38 by two isoforms of carboxylesterases (CES1 and 2) and butyrylcholinesterase in the human body [8, 10]. CES1 and CES2 are localized in small intestine, liver, colon, kidney, and blood cells, while butyrylcholinesterase is mainly found in plasma [11]. Formation of SN-38 by these esterases predominately occurs intra-hepatically and is a slow process, as only 2–5% of SN-38 is formed [8, 12]. At physiological pH, the lactone-ring of IRT and SN-38 can be hydrolyzed to a carboxylate isoform. As only the lactone form has antitumor activity, a small change in pH could alter the pharmacokinetics and efficacy of irinotecan [13]. In plasma, the carboxylate form of irinotecan and the lactone form of SN-38 dominate [14, 15]. Both IRT and SN-38 undergo carrier-mediated transported by the ABC transporter proteins: ABCB1, ABCC1, ABCC2, and ABCG2 [16].

The pathophysiology of IRT and SN-38-induced diarrhoea is complex and multifactorial, involving direct epithelial damage, pro-inflammatory mediator release, and alterations in gut microbiota [9, 17, 18]. Conventional antidiarrheal agents provide only limited relief, highlighting the need for an improved understanding of the underlying mechanisms. Following i.v. administration, IRT is extensively distributed to various tissues (volume of distribution > 240–400 l) and enzymatically converted to SN-38, by carboxylesterases, particularly carboxylesterase 2 (hCE2). hCE2 is highly expressed in the small intestine, colon, and liver, with the highest expression levels in the epithelium of the duodenal segment [7, 19]. It is primarily an intracellular enzyme, which is predominantly found in the endoplasmic reticulum of cells [20]. However, despite these highly generated levels, the effects of SN-38 in the duodenal segment are not well known [21]. In the distal small intestine and in the colon, IRT treatment is associated with a reduction in mucin-producing goblet cells alongside increased mucin secretion, likely due to altered mucin gene expression [22]. Additional damage to the enteric nervous system, including a decrease in enteric ganglia within the myenteric plexus, has also been reported [23]. Understanding these mechanisms is essential for developing strategies to mitigate IRT-induced GI toxicity and improve patient outcomes.

Proximal duodenal epithelial cells secrete bicarbonate at high rates to protect the mucosa from acidic gastric contents. Together with bicarbonate from the pancreas, this secretion establishes a near-neutral intraluminal environment required for activation of digestive enzymes [24–26]. Luminal pH may also influence the stability of certain drugs. For example, irinotecan (IRT) and its active metabolite SN-38 exist in a pH-dependent equilibrium between a lactone and a carboxylate form, a process largely driven by hydroxide ions. Because only the lactone form has antitumor activity, even small changes in pH may shift this equilibrium and thereby alter the pharmacokinetics and therapeutic efficacy of IRT and SN-38 [13]. In addition, proper regulation of duodenal epithelial paracellular permeability contributes to mucosal barrier function by preventing harmful luminal agents from entering the tissue and systemic circulation through this route [27]. This duodenal permeability is primarily regulated by epithelial apical tight junctions, which can rapidly reorganize in response to various physiological and/or pathophysiological stimuli [27]. The blood-to-lumen clearance of radiolabelled inert probe-markers of various molecular weight, such as ^51^Cr-EDTA or ³H mannitol, is well-established for measuring fast and continuous changes paracellular permeability, i.e. epithelial barrier function [28–34]. In living animals, we have previously shown that duodenal permeability shifts significantly with luminal perfusion of ethanol and acid [35]. Our group also recently showed that enteric intramural excitatory neural reflex involving both nicotinic and muscarinic receptor subtypes mediate an increase in mucosal permeability induced by luminal hypotonicity in rats [28]. Dysregulated tight junction proteins are linked to intestinal mucosal leakage and intestinal mucositis, and may be potentiated by increased intestinal motor activity [27].

The dynamic GI tract absorbs nutrients and fluids while acting as a protective barrier against harmful luminal substances is best reflected by the rat GI system. This in vivo pre-clinical model consists of a single layer of intestinal epithelial cells (IEC) covered by mucus and supported by an underlying immune system. The mucus layer is the first defence, separating microbiota from the epithelium. The IEC layer, mainly enterocytes and colonocytes, balances absorption and protection. These processes are regulated by region-specific transcriptional programs that drive the differentiation of specialized epithelial cell types, as well as by neuroendocrine feedback mechanisms that integrate luminal signals with epithelial and immune responses. Additionally, the gut-associated lymphoid tissue, the body’s largest immunological tissue, serves as a second-line defence system [36, 37].

The objective of this study is to examine whether administration of IRT induce changes in duodenal functions in the rat, in order to find possible targets for reducing intestinal toxicities in cancer patients. This is of particular interest, as this tissue is exposed for a range of physiological pH that has not previously been considered IRT GI toxicity. The specific aims were the following: (a) to characterize the effects of i.v. administered IRT on duodenal mucosal permeability and motility; (b) to characterize the dose-related impact of IRT on small and large intestinal epithelial morphology and faecal water content 72 h after administration; and (c) to monitor changes in cell viability during IRT and SN-38 exposure.

## Materials and methods

### Ethical statement

All experiments in this study were performed in strict accordance with the Guide for the Care and Use of Laboratory Animals of the National Institutes of Health and were approved by the local ethics committee for experiments with animals (permit 5.8.18–00250/2022) in Uppsala, Sweden. This study follows the ARRIVE guidelines and the study protocol established before the study included information on rat ID and weight, dose(s), dosing time and volumes, sampling times, samples, and sample handling [38]. A humane end-point was defined and animal well-being was monitored twice daily to avoid unnecessary suffering among the included animals. A body weight loss of > 20% was set as a criterion for early termination of the study.

### Chemicals and drugs

The anaesthetic 5-ethyl-5-(1’-methyl-propyl)-2-thiobarbiturate (Inactin^®^) purchased from Sigma-Aldrich (St. Louis, MO, USA). Sodium chloride (NaCl) and Resazurin sodium salt were purchased from Merck (Darmstadt, Germany). Irinotecan Actavis (solution for infusion 20 mg/ml), Irinotecan Fresenius Kabi (solution for infusion 20 mg/ml) and Voltaren^®^ (diclofenac sodium, solution for infusion 25 mg/ml) were purchased from Apoteket AB (Sweden). Irinotecan’s active metabolite 7-ethyl-10-hydroxycamptothecin (SN-38) was purchased from MedChemExpress (Monmouth Junction, NJ, USA). ^3^H-mannitol was purchased from PerkinElmer Life Sciences (Boston, MA, USA). Ethanol was purchased from Solveco AB (Rosersberg, Sweden). Xylene was purchased from VWR International S. A. S (Rosny-sous-Bois, France). DPX mounting medium, Pertex^®^ was purchased from Histolab^®^ (Askim, Sweden). Hematoxylin mayer and eosin were purchased from Histolab^®^ (Västra Frölund, Sweden). Dulbecco’s Modified Eagle Medium (DMEM), fetal bovine serum (FBS) and penicillin-streptomycin are purchased from ThermoFisher (Stockholm, Sweden).

### Animals

Male Sprague-Dawley rats weighing 250 ± 50 g was obtained from Taconic, Ejby, Denmark, and were delivered to the animal facility at the Biomedical Centre (BMC) at Uppsala University, Sweden. The animals were maintained under standardized temperature and light conditions (12:12-h light-dark cycle; temperature, 21–22 °C). The rats were acclimatized in the Animal Department for at least one week before experiments and were kept in enriched caged in groups of two or more with access to tap water and chow (R36, Lantmännen, Kimstad, Sweden) *ad libitum*.

Sample size was determined based on a power calculation for detecting a biologically relevant difference between groups using a two-sided test with α = 0.05 and 80% power. Assuming a large effect size typical for controlled intestinal perfusion experiments and reduced variability due to within-animal normalization, the estimated required sample size was approximately five animals per group. To ensure adequate power while adhering to the principles of reduction in animal experimentation, six animals per group were included.

### Experimental and surgical procedure for evaluating acute effects of IRT administration in vivo

Experiments were started by anesthetizing the animal with Inactin^®^ 120 mg·kg^− 1^ body weight intraperitoneally (i.p.). To minimize preoperative stress, anaesthesia was performed within the Animal Department at Uppsala University (UU), Sweden, by the same person who had previously handled the animals. Then, the rat was immediately transported to the laboratory for surgical procedure within the same building at UU.

At the laboratory, the animals were tracheotomized with a flexible tube to facilitate respiration, and the body temperature was maintained at 37.0 ± 0.5 °C throughout the experiments using a heating pad controlled by a rectal thermistor probe. The surgical and experimental procedures have been described previously [28, 39, 40]. A brief summary and some modifications are described here.

A femoral artery and a femoral vein were cannulated with PE-50 polyethylene catheters (Beckton Dickinson and Company, Sparks, MD, USA). The arterial catheter containing 20 IU/mL heparin isotonic saline was used for blood sampling and continuous systemic arterial pressure recordings via connection to a pressure transducer operated PowerLab^®^ system (AD Instruments, Hastings, UK). The vein catheter was used for continuous infusion of ^3^H-mannitol (paracellular mucosal permeability marker) in a 1.5 µCi/mL concentration at a rate of 1.0 mL/h, as well as for drug administration.

A laparotomy was performed, and the common bile duct was catheterized with a PE-10 polyethylene tubing close to its entrance into the duodenum (2–3 mm) to prevent pancreatico-biliary juice from entering the duodenum. Soft silicone tubing (Silastic^®^, Dow Corning, 1 mm ID) was introduced into the mouth, pushed gently along the oesophagus, guided through the stomach and pylorus, and secured by ligatures 2–5 mm distal to the pylorus. PE-320 tubing was inserted into duodenum approximately 2.5–3.5 cm distal to the pylorus and secured by ligatures. The proximal duodenal tubing was connected to a peristaltic pump (Gilson Minipuls 3, Villiers, Le Bel, France), and the segment was continuously perfused with a 37 °C 154 mM sodium chloride solution (saline) at a rate of ~ 0.4 mL/min. To complete the surgery, the abdominal cavity was closed with sutures, and the wound was covered with plastic foil.

^3^H-mannitol was administered intravenously as a bolus (0.2 mL, 3.0 µCi/mL) at this time, and as a continuous infusion of a 1.5 µCi/mL concentration at a rate of 1.0 mL/h. Voltaren i.v. injection 10 mg/kg was given 30 min after completion of the surgery to reverse the surgery-induced paralysis of the intestine [41]. After surgery, 60 min was allowed for the cardiovascular, respiratory, and intestinal function to stabilize before the experiments were commenced.

### Measurement of duodenal motility

Intraluminal pressure measurement was used to assess the duodenal wall contractions. The inlet perfusion tubing was connected, via a T-tube to a pressure transducer, and intraluminal pressure was recorded on a computer. The outlet tubing was positioned at the same level as the inlet tubing. An upward deflection of at least 2 mmHg above baseline was defined as a motor response. Changes in intraluminal pressure were recorded via a digitizer on a computer using PowerLab^®^ and the software Labchart7^®^ (ADInstruments Ldt. Hastings, East Sussex, UK). Duodenal motility was assessed in intervals of 10 min by measure of the total area under the pressure curve (area under the curve; AUC) during the sample period, as described previously [34]. The values given are mean ± SEM of three 10-min intervals (unless stated otherwise).

### Measurement of duodenal mucosal paracellular permeability

After the completion of the surgery, the administered radioactive isotope was permitted one hour for tissue equilibration to be achieved. The paracellular permeability of the duodenal segment was assessed by the blood-to-lumen clearance of ^3^H-mannitol (a sugar marker molecule that is metabolically stable in rats). Two blood samples (~ 0.2 ml each) were collected; the first was collected ten minutes before starting the experiment (at t= -10 min), and the second, at the end of the experiment (at t = 120). Each blood sample were centrifuged (5000 x g, 5 min) within ten minutes of sample collection, and 50 µL of the resulting plasma was collected for immediate analysis.

The duodenal segment was perfused with saline at a rate of 0.4 ml⋅min^− 1^, and the perfusate was quantitively collected at 10-min intervals. The ^3^H-activity of mannitol in each blood and perfusate sample was analysed using scintillation (Tri-Carb 2910 TR, Perkin Elmer Life Science, Boston, MA, USA). A linear equation analysis was performed on each sample to obtain a corresponding plasma value for each perfusate sample, to compensate for omission of repeated blood sampling throughout data collection. The blood-to-lumen clearance of ^3^H-mannitol was calculated as described previously [42] (Eq. 1), and expressed as (ml·minˉ^1^·100 gˉ^1^).

$$\:{}^{3}H-mannitol\:clearance=\:\frac{perfusate\:cpm\:x\:perfusion\:rate}{plasma\:cpm\:x\:wet\:tissue\:weight}x\:100$$

Equation 1: Equation used to determine duodenal mucosal paracellular permeability.

### Experimental protocol

In all of the experiments, the rates of duodenal mucosal paracellular permeability (ml·min^− 1^·100 g^− 1^), motility (AUC·10 min^− 1^), as well as systemic arterial blood pressure (mmHg) were monitored continuously and recorded at 10-min intervals. Control experiments were performed by measuring the parameters above during 120-min perfusions of the duodenal segment with isotonic saline (154 mM NaCl, 300 mOsm·kg^− 1^) at a rate of ~ 0.4 ml·min^− 1^.

In the animals administered irinotecan (IRT), the experiments started with a duodenal perfusion of saline for 30 min to collect basal data. Following this, an intravenous (i.v.) bolus infusion of IRT (60 mg/kg) was administered over a five-minute period. Parameters were collected for another 90 min, for a total of 120 min of data collection. Control animals were continuously perfused with saline/^3^H-mannitol cocktail for the full 120 min of experimental protocol to determine baseline control measurements and provide a means of comparison with IRT animals.

### Experimental procedure for evaluating effects of IRT 72 h after i.p. administration in vivo

#### Study design

This in vivo study segment initially comprised three groups of animals (*n* = 8 per group): a saline-treated control group and two treatment groups receiving a single intraperitoneal (i.p.) injection of IRT at 180 or 200 mg/kg. The final number of observations varied among experimental groups and outcome measures due to attrition and sample loss during tissue collection, histological processing and staining. The doses of IRT were selected based on previous experience and for clinical translation based on standard doses for IRT where we know that the bioavailability was high [42]. The rats also received a subcutaneous (s.c.) injection of atropine (0.02 mg) to avoid the immediate and short transient cholinergic side effects of IRT. At 72 h after these administrations, the experiments were terminated and the rats were anesthetised (Inactin^®^, 180 mg/kg). The full colonic content was retrieved, weighed and dried, while duodenal, jejunal, ileal, and colonic tissue samples were taken for morphological evaluation. The anaesthetized rats were then sacrificed by an intravenous injection of saturated potassium chloride.

#### Determining fecal water content

The faecal water content was measured by extracting the faecal content in colon followed by dehydration of the content. The body weight change was calculated from weight at t = 0 and t = 72.

#### Histology

Following fixation in 10% neutral-buffered formalin for 24 h, tissues were longitudinally opened and stored in 70% ethanol. Subsequently, samples underwent dehydration through a graded ethanol series, clearing with xylene, and paraffin infiltration using an automated tissue processor (STP 120 Spin Tissue Processor). Processed tissues were then embedded in paraffin blocks. Paraffin-embedded tissues were sectioned at 5 μm using a rotary microtome (Cool-Cut HM 255 S, Thermo Fisher Scientific, Waltham, MA, USA). Sections were mounted on glass slides (Corning^®^, NY, USA) and dried overnight on a heated surface.

Hematoxylin and eosin (H&E) staining was performed following standard protocols [43]. Briefly, sections were deparaffinized, rehydrated through graded alcohols, stained with Mayer’s hematoxylin, rinsed, counterstained with eosin, dehydrated, cleared, and coverslipped using DPX mounting medium. After drying overnight, stained sections were examined under a microscope, and images were captured using Zeiss Zen software. Morphometric analyses of villi and crypts were conducted using ImageJ software (National Institutes of Health, USA). For each animal, 10 villi and 10 crypts were measured from each segment of the small intestine, and 10 crypts were measured from the colon. To minimise selection bias, the villi and crypts were selected consecutively and measured systematically from left to right in each image. The mean villus and crypt lengths were then calculated for each animal and intestinal segment. Representative images were obtained using a Carl Zeiss AX10 microscope and the ZEN 3.2 microscopy software from ZEISS.

### Experimental procedure for evaluating concentration-response effects of IRT on cell viability

HCT116 cells (kindly gifted from Mårten Fryknäs, Uppsala University) were cultured in Dulbecco’s Modified Eagle Medium (DMEM) supplemented with 10% fetal bovine serum (FBS), and 1% penicillin-streptomycin. Cells were maintained at 37 °C in a humidified atmosphere with 5% CO₂. Subculturing was performed at 70–80% confluence using 0.25% trypsin-EDTA, with a seeding density of 2–4 × 10⁴ cells/cm². For the concentration-response study, cells were seeded in 96-well plates at a density of 7.5 × 10^4^ cells per well and allowed to adhere overnight. Subsequently, in parallel experiments performed under identical conditions, cells were exposed to either IRT or SN-38 at concentrations ranging from 0.001 to 100 µM for 48 h. Control wells received vehicle treatment only. Cell proliferation was monitored via a resazurin reduction assay. A 1% resazurin sodium salt solution (R7017-1G, Sigma–Aldrich, Darmstadt, Germany) was added in 1/80 dilution to the cells and incubated for 6 h, after which absorbance was measured at 570 and 600 nm on a BioTek Synergy H4 Multi-Mode plate reader.

### Statistical analysis

In vitro experiments were done in at least three biological replicates, which we define as parallel measurements of biologically distinct samples taken from independent experiments. Technical replicates we define as loading the same sample multiple times on the final assay. The in vivo experiments were done on at least six independent animals. Outliers were kept in the analyses, unless they were suspected to occur due to technical errors, in which case the experiment was repeated. Values are expressed as means ± SEM, with the number of experiments given in parentheses. The statistical significance of the data was tested by repeated measures analysis of variance (ANOVA). To test the differences within a group, a one-factor repeated measures ANOVA was used, followed by a Tukey post-hoc test. Between groups, a two-way repeated measures ANOVA was used, followed by a Bonferroni post-hoc test.

All the statistical analyses were performed using GraphPad Prism 10.4 software (GraphPad Software Inc., San Diego, CA, USA). A *P-*value less than 0.05 was considered significant.

## Results

### Acute effects on IRT intravenous (i.v.) administration in vivo

In control animals perfused with isotonic saline the mean arterial blood pressure (MABP) was 91 ± 2.5 mmHg (not shown). In the irinotecan (IRT) group the MABP was 79 ± 3 mmHg (not shown). It should be noted that administration of IRT (at t = 30 min) induced a 5–10 min transient decrease in MABP (~ 10–15 mmHg), a value that stabilised slightly below the previous baseline value.

#### Duodenal mucosal paracellular permeability

**Fig. 1:**
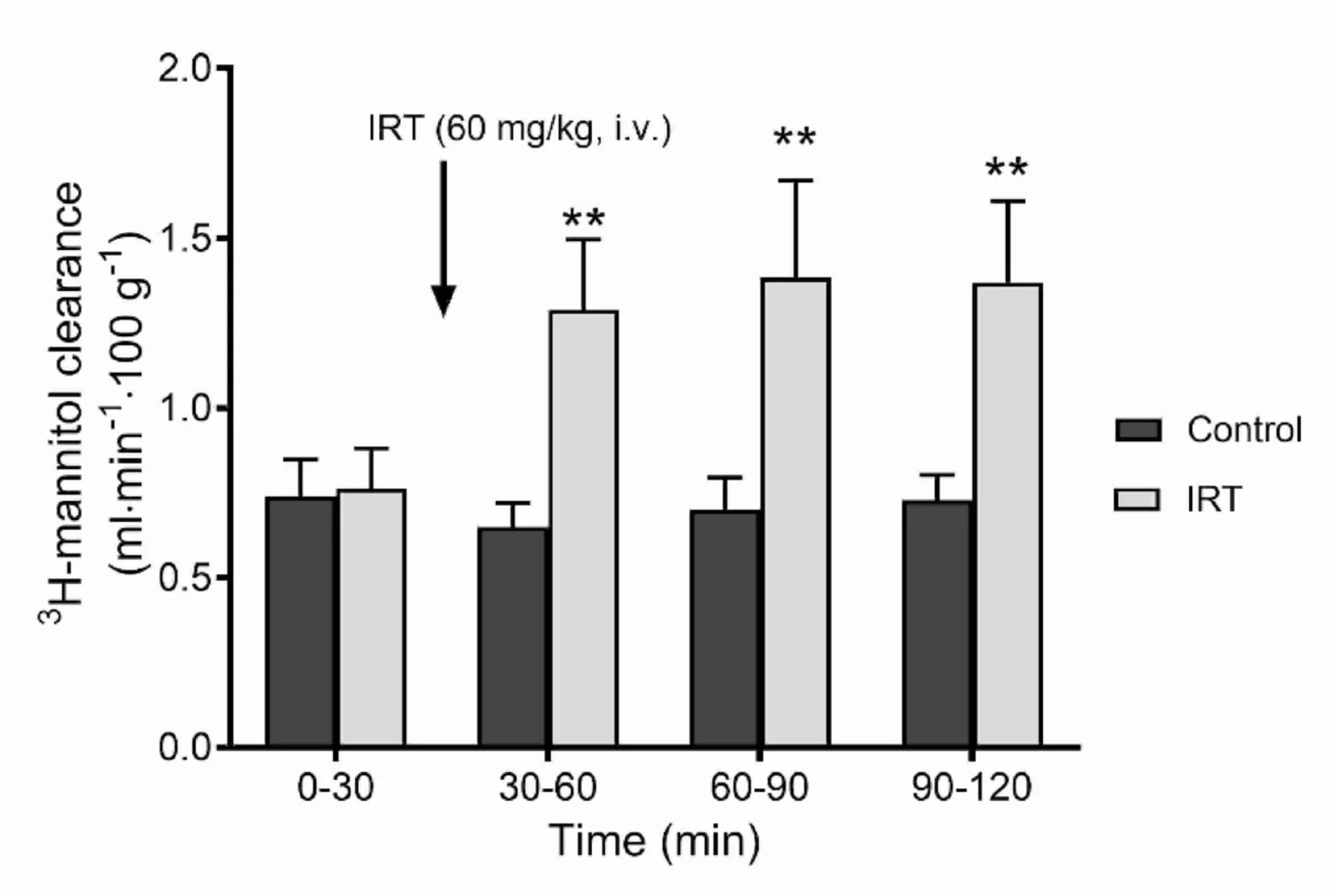
Effects of IRT on duodenal epithelial paracellular permeability. Administration of irinotecan (IRT) (60 mg/kg, i.v.) caused a robust and sustained increase in duodenal epithelial blood-to-lumen clearance of ^3^H-mannitol. In controls the permeability remained stable throughout experiment. Values are mean ± SEM. ** indicates a significant (*P* < 0.01) difference compared with the control group

In control animals perfused with isotonic saline, the duodenal mucosal paracellular permeability was stable during the 120 min experimental period. At the start of the experiment, duodenal permeability, calculated as duodenal clearance of mannitol in Eq. 1, was 0.74 ± 0.11 and at the end 0.73 ± 0.07 ml·min^− 1^·100 g^− 1^; *n* = 6, *P* > 0.05; (Fig. 1). To investigate the acute effect of IRT treatment on duodenal paracellular permeability, an i.v. injection of IRT at a dose of 60 mg/kg was given. IRT dosing induced an immediate and sustained increase in duodenal paracellular permeability significant from baseline, from 0.76 ± 0.12 to 1.37 ± 0.24 ml·min^− 1^·100 g^− 1^; *n* = 6, *P* < 0.01; Fig. 1).

#### Duodenal motility

**Fig. 2:**
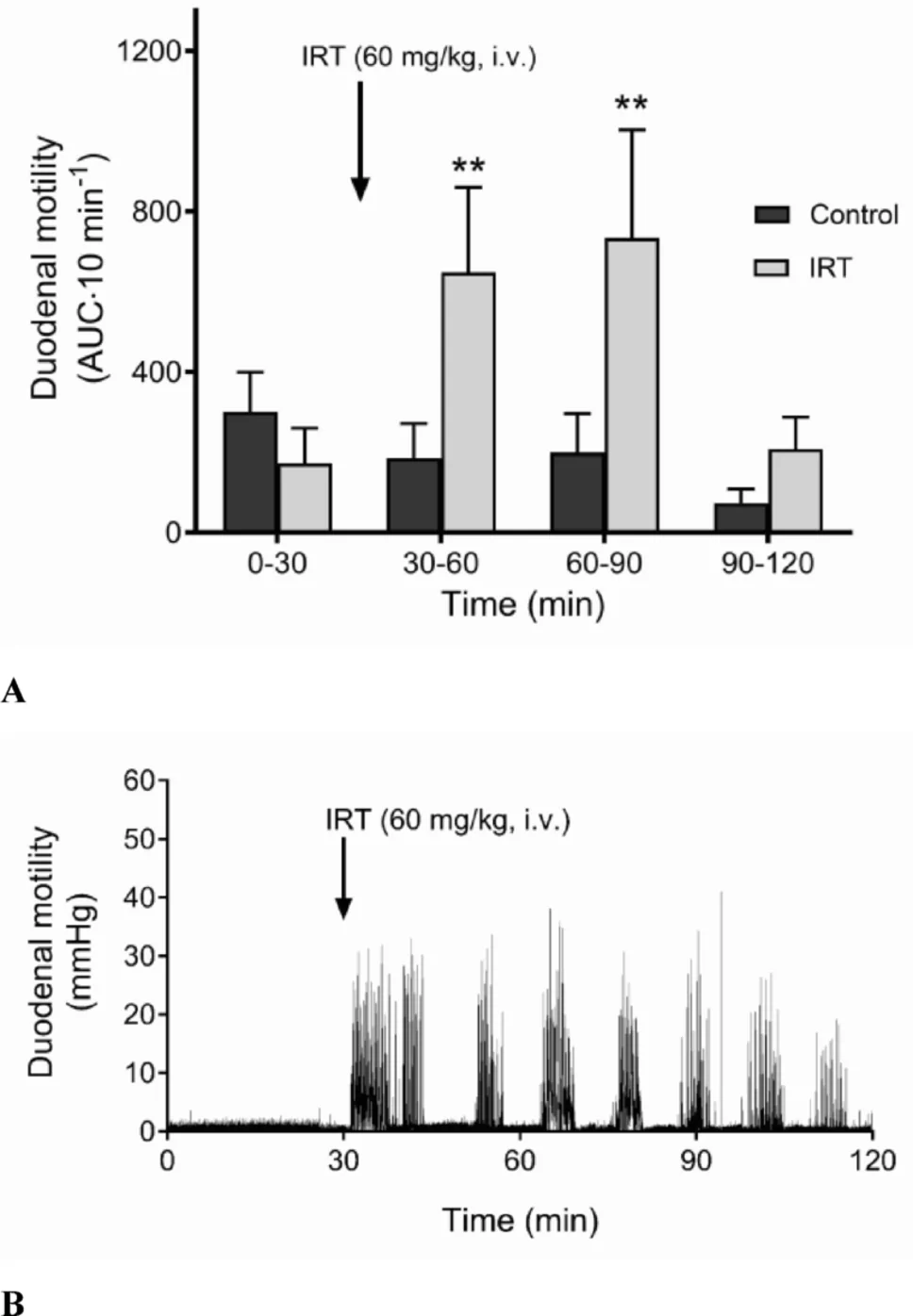
Effects of IRT on duodenal motility. **A**) Administration of irinotecan (IRT) (60 mg/kg, i.v.) caused a pronounced transient increase in duodenal motility. 60 min post IRT administration motility returned to the baseline value. In controls the motility stable with a slight decrease throughout experiment. Values are mean ± SEM. ** indicates a significant (*P* < 0.01) difference compared with the control group. **B**) IRT (60 mg/kg bolus i.v.) induce an immediate increase in duodenal motility within 5 min after administration. The figure illustrates a representative recoding from one experiment

In controls, the duodenal motility slightly declined during the 120 min experimental period, from 301 ± 99 to 73 ± 35 AUC·10 min^− 1^, values that was not significantly different (*n* = 6, *P* > 0.05; Fig. 2A). Administration of IRT (60 mg/kg bolus i.v.) induce an instant increase (Fig. 2B) in duodenal motility from 172 ± 87 to 648 ± 212 AUC·10 min^− 1^, and continued to increase over the first 60 min to a maximum value of 733 ± 269 AUC·10 min^− 1^ (*n* = 6, *P* < 0.01), Fig. 2A. During the last 30 min of the experiment motility returned to a value not significantly different from the initial baseline value (208 ± 80 AUC·10 min^− 1^ (*P* > 0.05).

### Effects of IRT 72 h after intraperitoneal (i.p.) administration

#### Histological evaluations

**Fig. 3:**
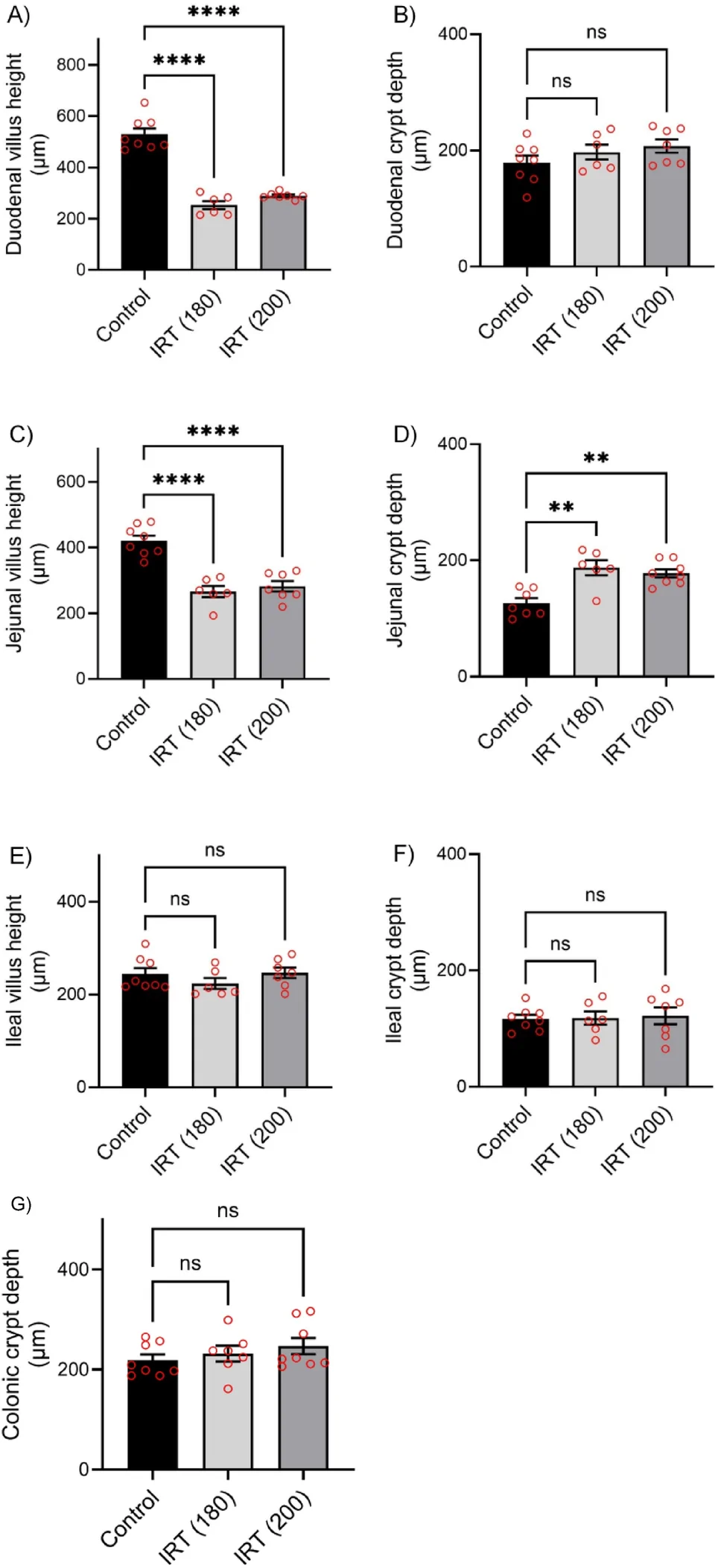
Effects of IRT on intestinal villus length and crypt depth 72 h post i.v. administration. Effects of irinotecan (IRT) (180 mg/kg and 200 mg/kg administered i.p.) on **A**) duodenal villus length, **B**) duodenal crypt depth, **C**) jejunal villus length, **D**) jejunal crypt depth, **E**) ileal villus length, **F**) ileal crypt depth, and **G**) colonic crypt depth. Values are mean ± SEM. ** and **** indicates significant (*P* < 0.01 and *P* < 0.0001) difference compared with the control group

The small intestinal villus height and crypt depth in rats was assessed following a single i.p. injection of a dose of IRT (180 mg/kg, *n* = 6) or IRT (200 mg/kg, *n* = 7). Samples were taken at 72 h post-dosing. The villus height (from villus tip to villus–crypt junction) and crypt depths (defined as invagination depth between adjacent villi) were determined. Histological assessment revealed that a single i.p. dose of IRT (180 mg/kg) or IRT (200 mg/kg) significantly reduced the duodenal villus height from 529 ± 23 μm (control, *n* = 8) to 253 ± 16 μm and 289 ± 5 μm, respectively (*p* < 0.0001), as shown in Fig. 3A. Neither IRT (180 mg/kg, i.p.) nor IRT (200 mg/kg, i.p.) significantly changed the duodenal crypt depth, Fig. 3B.

**Fig. 4:**
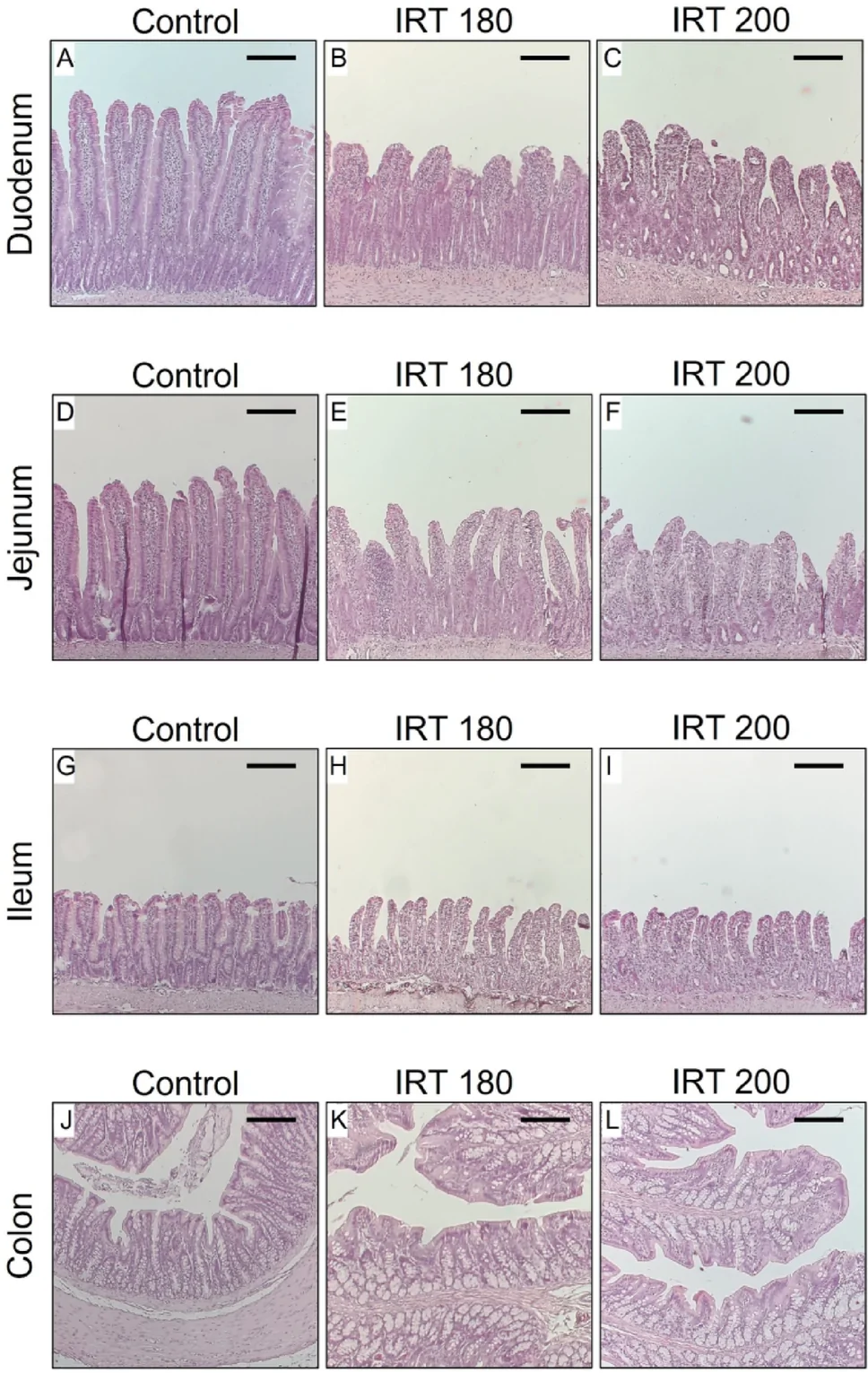
Hematoxylin-eosin staining of the rat intestine. Different specimens from the morphological examination after hematoxylin-eosin staining of the rat intestine 72 h post i.p. administration with saline or irinotecan (IRT) in doses of 180 mg/kg or 200 mg/kg. Representative images are shown for the duodenum in (**A**-**C**), jejunum in (**D**-**F**), ileum in (**G**-**I**), and colon in (**J**-**L**). The scale indicates 200 μm

Similarly, IRT (180 mg/kg) and IRT (200 mg/kg) significantly reduced jejunal villus height from 420 ± 16 μm (control, *n* = 8) to 266 ± 17 μm and 282 ± 16 μm, respectively (*p* < 0.0001), as shown in Fig. 3C. IRT (180 mg/kg) and IRT (200 mg/kg) significantly increased jejunal crypt depth, from 126 ± 9 μm (control, *n* = 8) to 187 ± 13 μm and 178 ± 7 μm, respectively (*p* < 0.01), as shown in Fig. 3D. IRT (180 mg/kg) and IRT (200 mg/kg) did not affect ileal villus height (Fig. 3E), ileal crypt depth (Fig. 3F), or colonic crypt depth (Fig. 3G). Representative images are shown for the duodenum (Fig. 4A-C), jejunum (Fig. 4D-F), ileum (Fig. 4G-I), and colon (Fig. 4J-L).

#### Colonic faecal water content

**Fig. 5:**
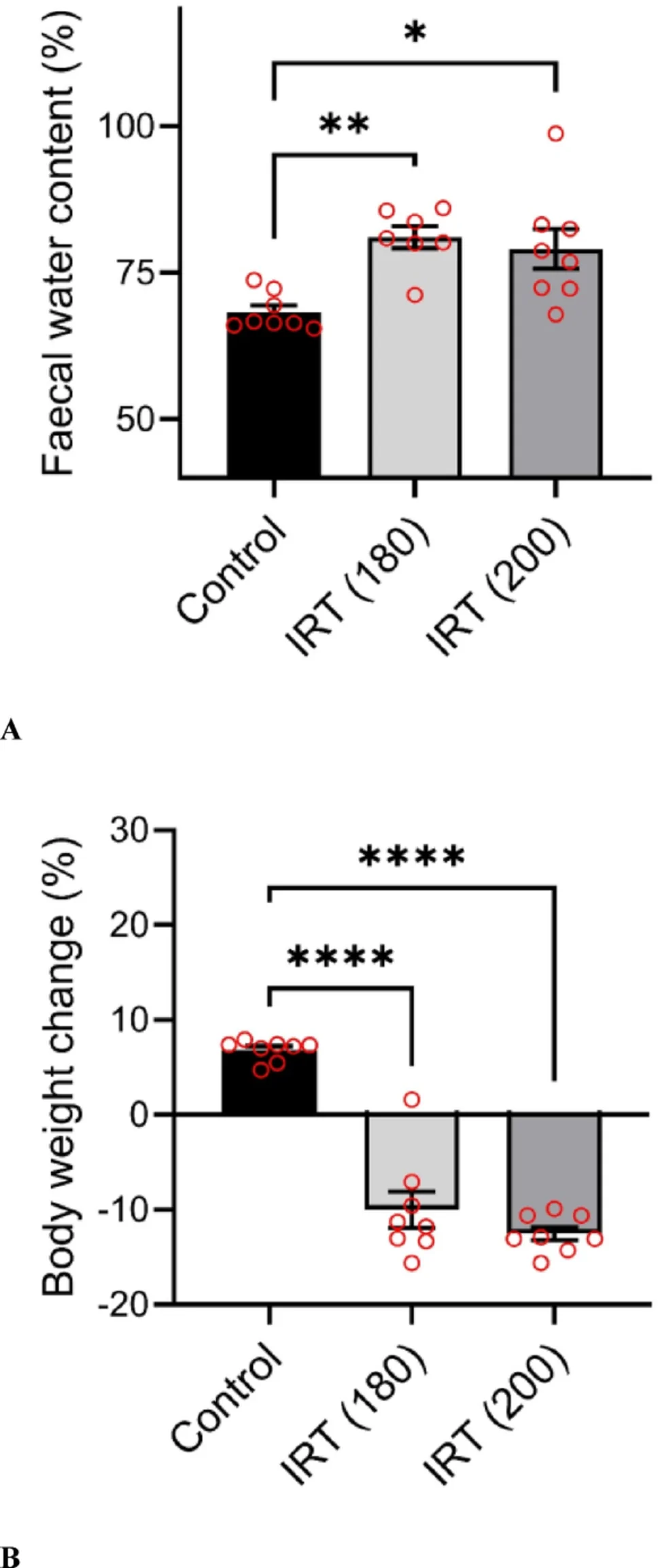
Effects of IRT on faecal water content and body weight. **A**) Colonic faecal water content in % and, **B**) change in body weight, 72 h after dosing with irinotecan (IRT) i.p. in doses of 180 mg/kg or 200 mg/kg. Values are mean ± SEM. * and ** indicates significant (*P* < 0.05 or *P* < 0.01, respectively) difference compared with the control group

The degree of diarrhea was determined by collecting fecal contents of the colon into plastic tubes. After drying, the water contents could be quantified, calculated and expressed as percent of water (%H_2_O). The results at 72 h after i.p. administration showed that both IRT (180 mg/kg) and IRT (200 mg/kg) increased the fecal water contents from 68 ± 1.1% (control animals) to 81 ± 1.9% and 79 ± 3.4%, respectively (*p* < 0.01) (Fig. 5A).

#### Changes in body weight

Animals given IRT i.p. (180 mg/kg) and IRT (200 mg/kg), demonstrated a significant reduction in body weight 72 h after administration, -10.1 ± 1.9% and − 12.5 ± 0.7% respectively, compared to controls (+ 6.8 ± 0.4%) (Fig. 5B).

### In vitro – concentration-response effects of IRT and SN-38 on cell viability

**Fig. 6:**
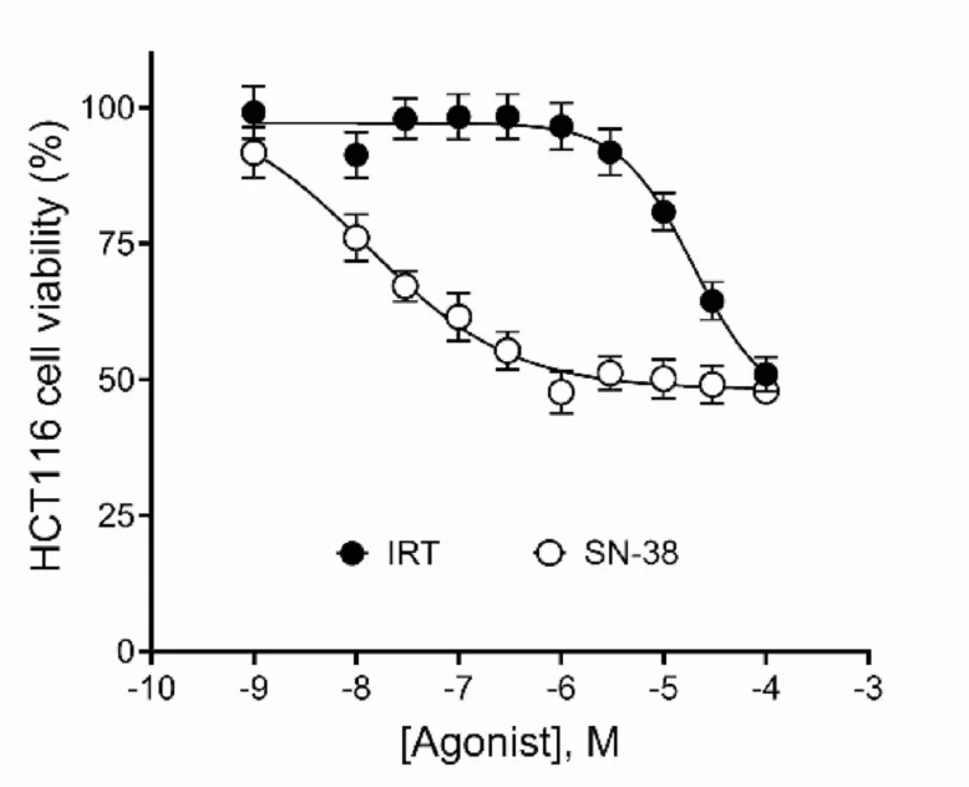
Effects of IRT and SN-38 on HCT116 cell viability. Graph representing % cell viability versus logarithm of the concentration of IRT and SN-38. IC_50_ was calculated to 19.4 µM for IRT and to 12.8 nM for SN-38

HCT116 cells were exposed to IRT and SN-38 at concentrations ranging from 0.001 to 100 µM, and cell viability was assessed using the resazurin reduction assay. The effect of IRT and SN-38 on cell viability was concentration-dependent, resulting in a calculated IC₅₀ value of 19.4 µM for IRT and of 12.8 nM for SN-38 (Fig. 6). These findings confirm that IRT and SN-38 exerts concentration-dependent cytotoxic effect on intestinal epithelial cells in vitro, and that the active metabolite of IRT, SN-38 has approximately a 1000-fold higher potency and is a more effective inhibitor than IRT when used in in vitro systems.

## Discussion

Chemotherapy-induced intestinal toxicity is a serious side effect that leads to significant disruptions in gut function and has a significant negative impact of quality-of-life of cancer patients, which may also reduce the adherence to medical therapy [44, 45]. It is marked by increased intestinal permeability, inflammation of the mucosa, digestive impairments, fluid imbalance, and difficulties in absorbing electrolytes and nutrients [3, 4]. Additionally, it can cause microbial imbalances (dysbiosis) and changes in intestinal motility, resulting in a range of GI symptoms and broader systemic health complications [4, 36, 46]. There is a great medical need to find innovative treatments that can be developed into clinical strategies for managing chemotherapy-induced intestinal toxicity [47, 48]. However, the fundamental insights gained from these pre-clinical dynamic models with its intact neuroendocrine signalling pathways has a key role in improving our current understanding of the pathophysiology, prevention, and treatment of intestinal toxicity.

For the first time, we demonstrate that IRT and its formed active metabolite SN-38 induces distinct and interconnected effects on intestinal physiology in such a highly dynamic model, linking its acute impact on duodenal motility and mucosal barrier function with delayed structural damage and increased faecal water content. As the duodenal tissue is exposed for a physiological range of GI pH and the conversion of carboxylate and lactone form of both IRT and SN-38 is pH sensitive this add a new knowledge. Firstly, IRT administration in rats leads to significant increases in duodenal mucosal paracellular permeability and motility, which occurred within minutes after i.v. injection. Additionally, one important in vivo observation included an IRT-induced villus atrophy predominantly in the proximal small intestine, as assessed 72 h after a single i.p. dose. One possible explanation is that the duodenal epithelium has a high enzymatic capacity of carboxylesterase 2 to metabolize the prodrug IRT to form the active substance SN-38 [21, 49], together with a local pH profile that may favour the conversion of the lactone forms of IRT and SN-38. However, the present study was not designed to directly investigate these enzymatic-mediated mechanisms.

Furthermore, we found that IRT treatment results in increased faecal water content, consistent with previous studies [9, 50–55]. Altogether, these observations collectively highlight the multifaceted impact of IRT treatment on gut function, providing new insights how the mechanisms and their timing contribute to chemotherapy-induced intestinal toxicity.

Intestinal paracellular permeability, assessed by ³H‑mannitol clearance, is governed by tight junctions within the apical junctional complex [23, 45]. These junctions form dynamic, selective pores that regulate passive transport of small solutes (< 200 Da) and fluid across the epithelium, making the paracellular route the major rate‑limiting pathway [27]. To our knowledge, no continuous measurement has been reported of in vivo duodenal permeability of low-molecular-mass probes following administration of IRT. These measurements contribute in filling a knowledge-gap on how IRT and SN-38 mediate mucosal injury and barrier dysfunction. In this study, we determined duodenal mucosal paracellular permeability by continuously monitoring the blood-to-lumen clearance of ^3^H-mannitol, a well characterized probe (182 g/mol) that gives a valid estimate on changes in epithelial paracellular permeability. Smaller probes, such as mannitol, are likely to be transported paracellularly along the whole crypt-villus axis [29]. After i.v. administration of IRT, an increase in duodenal permeability was observed within 5–15 min, suggesting that IRT causes dilation of the epithelial paracellular pathway. The duration of this increase in permeability remained high during the rest of the experiment. One possible explanation for the near instant increase in permeability, or leakage, is that IRT and/or rapidly formed SN-38 may directly or indirectly affect intracellular signaling pathways in intestinal enterocytes, leading to changes in intercellular adhesion. However, these signaling events were not examined in the present study and should therefore be regarded as a hypothesis.

The sustained increase in intestinal permeability may be related to epithelial damage, as previously reported toxic effects of IRT on intestinal epithelial cells have been associated with dysregulation of tight junctions. Supporting this, we observed a concentration-dependent cytotoxic effect of IRT on HCT116 cells, with a significant reduction in cell viability at increasing doses. Interestingly, previous studies on irinotecan’s cytotoxic effects on CaCo-2 cells, a human cell line (monolayer) with a colonic origin, have shown variability in cell survival outcomes based on concentration and exposure duration. A study by Kaku et al. (2015) reported that irinotecan does not induce apoptosis in CaCo-2 cells, but their experiments were conducted with a maximum concentration of 30 µM over 24 and 48 h, which is significantly lower than the concentrations used in our study [56]. Another in vitro cell study observed a 40% reduction in cell survival at approximately 170 µM and complete cell death at 1477 µM after 24 and 48 h [57]. However, in the present study we show that the active metabolite of IRT, SN-38, exerts concentration-dependent cytotoxic effects with an approximately a 1,000-fold greater potency and is a significantly more effective inhibitor than IRT when used in in vitro systems. Overall, this suggests that irinotecan’s cytotoxic effects are mainly mediated by SN-38 and are highly concentration-dependent, with significant variations in cell survival at different concentrations and likely also influenced by initial seeding densities and drug exposure times [57]. This loss of viability following in vitro exposure to SN-38 may provide support for the hypothesis that epithelial injury contributes to the observed increase in duodenal permeability in the in vivo rat model. However, because cell culture and in vivo experiments are two entirely different experimental systems, a direct mechanistic link cannot be established from these data. Together, these findings suggest that IRT and SN-38 not only induces transient functional changes in epithelial permeability, but may also contribute to epithelial injury, which may play a critical role in chemotherapy-induced mucosal toxicity.

Further supporting this, our previous studies have shown that duodenal mucosal paracellular permeability returns to baseline level after cessation of the duodenal perfusion with the potent barrier breaker ethanol (10–15%), indicating that the response on duodenal permeability is reversible when the toxicity does not induce damage [35]. Similar dynamics has been shown in vitro using CaCo-2 cell line, that noncytotoxic concentrations of ethanol increases paracellular permeability by displacement and disruption of tight junction proteins and that the paracellular gaps re-closed when ethanol was removed [58]. We have previously found that a single dose of IRT does not have any impact on jejunal mucosal paracellular permeability at a time-point of 72 h after i.p. administration, although the dose given was almost three times higher than in the present study (150 mg/kg i.p. vs. 60 mg/kg i.v.) [42]. This suggests that in healthy rodents, IRT treatment-induced increases in small intestinal permeability may be relatively transient following administration of a single dose. However, it remains to be investigated how repeated injections of IRT with recovery intervals would affect the small intestinal permeability, and how experimental cancer models, where both the presence of tumours and the effects of treatment may influence adverse outcomes, would alter these responses. It is also necessary to determine systemic and local intestinal tissue exposure of unchanged prodrug IRT and active drug SN-38 and their carboxylate and lactone forms that are locally formed by intracellular enzymes and through pH-dependent conversion in order to be able to establish a concentration-effect relationship for these effects for both prodrug and active drug. It is also unclear whether this rather short and transient increase in permeability is sufficient to initiate a sustained and self-perpetuating cycle of mucositis. This is a dynamic area that need further investigating in models with intact and complete neuroendocrine systems that keep of full feedback systems of all GI conditions.

Chemotherapy may influence GI motility either directly, through effects on smooth muscle cells, or indirectly, by altering the enteric nervous system. However, this remains an underexplored area of research [48]. While intestinal transit time can be obtained from animal models and patients, it provides only limited information on the local tissue and segment motility pattern. In contrast, experiments on anesthetized animals with an isolated intestinal segment, preserving both an intact blood supply, enteric neural network, and endocrine feedback systems, as used in the present study, offer an attractive experimental in vivo model where numerous functional mechanisms can be investigated simultaneously. An interesting finding in our study was that the duodenal motility was greatly affected by i.v. administration of IRT. The increase in motility had an almost immediate (within 5 min) onset after injection, an in vivo observation that has not been described previously, and may indicate that the prodrug compound IRT also contributes to this response. However, the relative contribution of IRT versus rapidly formed SN-38 cannot be determined from this study. This effect may, at least in part, contribute to the early GI symptoms reported in some cancer patients following clinical use [45], although the clinical relevance remains to be determined. This emphasizes the need to further clarify the role of IRT and SN-38 on these GI effects. Supporting this, previous studies have shown that IRT can induce early-onset diarrhoea as a consequence of its inhibition of acetylcholinesterase, to induce an acute cholinergic syndrome [9]. The increased levels of acetylcholine, may induce intestinal motor dysfunction due to stimulation of cholinergic muscarine receptors [5], which could explain the rapid increase in motility. However, conversely, we recently showed that administration of bethanechol, a specific muscarinic agonist without any effect on nicotinic receptors, did not affect the duodenal motility in the same experimental model [28]. These findings highlight the complexity of IRT-induced motility changes and warrant further investigation across different intestinal segments. While our study demonstrates increased intestinal motility following IRT i.v. administration, Boeing et al. have reported the opposite effect, showing reduced digestive motility in mice treated with IRT [59]. This apparent discrepancy underscores the need for further research to clarify the mechanisms underlying chemotherapy-induced alterations in intestinal motility by IRT and SN-38.

The different IRT doses used in the acute and delayed-effect in vivo experiments were chosen to reflect the distinct experimental objectives while maintaining physiological stability. In the acute studies, administering the higher dose of IRT (180 mg/kg, i.v.) induced a profound and sustained reduction in mean arterial pressure (MAP < 50 mmHg), with no recovery observed throughout the experimental observation period. Accordingly, a lower dose of IRT (60 mg/kg, i.v.) was selected for the acute experiments. This dose was sufficiently high to permit the detection of early changes in intestinal motility and epithelial paracellular permeability, while avoiding the profound hemodynamic instability observed at the higher doses. Thus, the use of two dose levels was a deliberate and necessary aspect of the experimental design, allowing the acute and delayed effects of IRT to be evaluated under conditions appropriate to each experimental endpoint while minimizing confounding effects of severe systemic toxicity.

In this study classical signs of intestinal toxicities were observed 72 h post-IRT i.p. administration, including body weight loss, increased fecal water content, and histopathological changes of the small intestine and colon. IRT treatment induced a marked shortening the duodenal and jejunal villi, while no differences in ileal villi length were observed.

A particularly interesting finding was the occurrence of jejunal crypt hyperplasia in rats sacrificed 72 h after i.p. IRT administration. This hyperplasia was not observed in any other segments. A similar hyperplasia was previously reported by Gibson et al. 2003 [60], and has been confirmed in rodents by others [53, 61], including our group [51]. One possible explanation for this phenomenon is that the jejunum exhibits an increase in mitotic cell numbers, potentially reflecting initiation of a recovery process following IRT-induced damage. However, cell proliferation was not directly assessed in this study. We found that morphological changes in the colonic epithelium were minimal. However, this does not necessarily mean that the function of the epithelium in the crypts in this segment are unaffected by IRT. Previous studies have shown an increase of colonic crypt 24 h after administration of doxorubicin (Dekaney et al., 2009).

Chemotherapy-induced diarrhoea can be a severe condition, potentially limiting treatment or posing serious health risks [4, 9]. This study’s findings align with previous research [53–55], demonstrating that IRT treatment leads to a significant rise in faecal water content, an established marker of diarrhoea in animal studies. Additionally, the rapid and substantial body weight loss observed within 24 h suggests that the decrease is primarily due to increased faecal output and reduced food and water intake, rather than a direct loss of fat or muscle mass. However, this remains speculative, as we did not measure these factors directly.

The increase in faecal water content during the first 24 h after administration is likely due to a combination of increased small intestinal motility and increased mucosal permeability. However, the available data suggest that diarrhoea may involve additional mechanisms beyond the acute changes in motility and permeability observed in this study. One proposed mechanism, established in animal models, involves the metabolism of SN-38. When SN-38 undergoes hepatic glucuronidation, it is converted into SN-38 glucuronide (SN-38G) and excreted into the intestinal tract via bile. In the colon, bacterial β-glucuronidases can hydrolyse SN-38G, reactivating SN-38 that causes damage to the epithelium when it is reabsorbed, which might contribute to delayed-onset diarrhoea [62].

## Conclusions

In conclusion, IRT markedly alters duodenal motility and mucosal barrier function, with increased motility and permeability likely contributing to both acute and prolonged diarrhea. The most pronounced histopathological changes occurred in the proximal small intestine, supporting the idea that early disruptions in motility and permeability drive IRT- and SN38-induced intestinal toxicity. Clarifying how epithelial fluid handling, electrolyte transport, permeability, and motility interact in the proximal intestine, where IRT is converted to SN 38, may guide strategies to improve efficacy and reduce toxicity.

## Data Availability

The datasets used and/or analysed during the current study are available from the corresponding author on reasonable request.
